# Validated HPTLC Method for Simultaneous Determination of Quinapril Hydrochloride and Hydrochlorothiazide in a Tablet Dosage Form

**DOI:** 10.4103/0250-474X.44612

**Published:** 2008

**Authors:** Girija B. Bhavar, V. A. Chatpalliwar, D. D. Patil, S. J. Surana

**Affiliations:** Department of Pharmaceutical Chemistry, R. C. Patel College of Pharmacy Karvand Naka, Shirpur, Dist: Dhule-425 405, India; 1 H.R. Patel College of Pharmacy Karvand Naka, Shirpur, Dist: Dhule-425 405, India

**Keywords:** HPTLC, quinapril hydrochloride, hydrochlorothiazide

## Abstract

Quinapril hydrochloride and hydrochlorothiazide were simultaneously determined by HPTLC in pharmaceutical formulations. The drugs were separated on silica gel 60 F_254_ plates using suitable combination of solvents as mobile phase. The validation parameters, tested in accordance with the requirements of ICH guidelines, prove the suitability of methods.

Quinapril hydrochloride (fig. 1), chemically, (3*S*)-2-[(2*S*)-2-[[(1*S*)-1-(ethoxycarbonyl)-3-phenylpropyl]amino]-1-oxopropyl]-1,2,3,4-tetrahydro-3-isoquinolinecarboxylic acid hydrochloride^1^, is an angiotensin converting enzyme (ACE) inhibitor, used to treat hypertension and cardiac failure^2^,^3^. Different HPLC methods have been reported for the analysis of quinapril and its active metabolites^4^,^5^. Quinapril has been separated from its metabolite, quinaprilat, from biological fluid using gradient elution-reversed phase chromatographic system^6^.

**Fig. 1:**
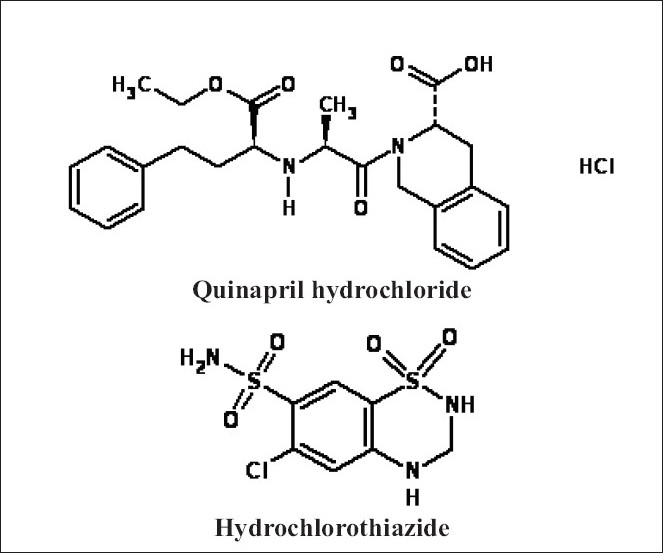
Structures of quinapril hydrochloride and hydrochlorothiazide

Hydrochlorothiazide (fig. 1), chemically, 6-chloro-3,4-dihydro-2*H*-1,2,4-benzothiadiazine-7-sulfonamide-1,1-dioxide^7^–^11^, is a thiazide diuretic used in the treatment of oedema associated with heart failure and with renal and hepatic disorders. Different analytical methods using HPTLC^12^, HPLC 13–18 and spectrophotometry^19^–^20^ have been reported for hydrochlorothiazide. However, there is no method reported for simultaneous estimation of quinapril hydrochloride and hydrochlorothiazide using HPTLC or HPLC. The proposed methods presented here are simple, fast, accurate and precise HPTLC methods for simultaneous determination of quinapril hydrochloride and hydrochlorothiazide in tablets.

Quinapril hydrochloride (batch no. NCL/QN/18) and hydrochlorothiazide (batch no.5005HCRJ) were supplied as a gift sample from Macleods Pharmaceuticals, India. All chemicals used were of HPLC grade, purchased from Qualigens fine Chemicals, Mumbai, and were used as such. The tablets were procured from local market.

Samples were applied (Hamilton microsyringe using Linomat V applicator, Switzerland) as 6 mm bands spaced 13 mm on 20 cm×10 cm aluminum backed silica gel 60 F_254_ TLC plates (Merck, Darmstadt, Germany). Development was achieved for 70 mm in Camag twin-trough chamber (20 cm×10 cm) previously saturated for 15 min at 25±2° with the vapors of solvent system ethyl acetate: acetone: acetic acid (6.5: 3: 0.5 v/v/v). Subsequently, plates were dried and scanned at 208 nm using Camag TLC scanner 3 equipped with Wincats software (version 1.3.0) and deuterium light source.

Standard stock solution containing 100 μg/ml of quinapril hydrochloride and 125 μg/ml of hydrochlorothiazide were prepared in methanol. Linearity was performed by applying six times the stock solution to give concentrations of 400-2800 ng/spot and 500-350 ng/spot of quinapril hydrochloride and hydrochlorothiazide, respectively. Calibration curve was established by plotting peak area on ordinate and corresponding concentration on abscissa.

Twenty film-coated tablets (Accuretic, Pfizer) were accurately weighed, and their average weight was determined. Powder equivalent to 10 mg of quinapril hydrochloride and 12.5 mg of hydrochlorothiazide was dissolved in methanol, sonicated for 20 min; solution was filtered and diluted to 100 ml with methanol. The solution was applied on the plate to give 1200 ng/spot of quinapril hydrochloride and 1500 ng/spot of hydrochlorothiazide, respectively. Six such plates were developed as described above. The results of assay are summarized in Table 1. The method was validated as per the various parameters given in ICH guidelines^21^.

**TABLE 1:** RESULTS OF ASSAY OF TABLETS

Tablet	Component	Label Claim (mg)	Amount Found mg±SD (n=6)	% Label Claim	% RSD
Batch 1	Quinapril hydrochloride	10	9.97±0.08	99.75	s0.78
	Hydrochlorothiazide	12.5	12.45±0.14	99.67	1.12
Batch 2	Quinapril hydrochloride	10	9.96±0.068	99.59	0.68
	Hydrochlorothiazide	12.5	12.39±0.11	99.16	0.87

n is the number of repetitions

The linearity was studied in the concentration range of 400–2800 ng/spot for quinapril hydrochloride and 500–3500 ng/spot for hydrochlorothiazide. Precision of the method is expressed in terms of% RSD.

The peak purity of quinapril hydrochloride and hydrochlorothiazide was determined by correlating the spectra’s of drug at the peak start (S), peak apex (M) and at peak end (E) positions. Recovery studies were performed by standard addition method at 80%, 100% and 120% level, to the pre-analyzed samples and contents were reanalyzed, using the proposed method. Ruggedness of the proposed method was determined by performing assay by two different analysts, using similar operational and environmental conditions.

Linearity was observed in the concentration range of 400 - 2800 ng/spot and 500 - 3500 ng/spot; correlation coefficients (r) being 0.9992 and 0.9993 with the slopes 2.094 and 1.8753 for quinapril hydrochloride and hydrochlorothiazide, respectively. The intra-day and inter-day precision ranged between 0.212-0.380% and 0.217-0.416% for quinapril hydrochloride and between 0.325-0.642% and 0.434- 1.162% for hydrochlorothiazide, respectively. LOD and LOQ were found to be 123.02 ng/spot and 372.77 ng/spot for quinapril hydrochloride and 143.82 ng/spot and 435.81 ng/spot for hydrochlorothiazide, respectively.

Specificity of the method was well demonstrated by efficient separation of both drugs by the solvent system (fig. 2). The R_f_ values for quinapril hydrochloride and hydrochlorothiazide were found to be 0.51 and 0.76, respectively. The peak purity is expressed as correlation r (S, M) = 0.9986, r (M, E) = 0.9967 for quinapril hydrochloride, and correlation r (S, M) = 0.9996, r (M, E) = 0.9995 for hydrochlorothiazide. The recoveries for quinapril hydrochloride and hydrochlorothiazide were ranged between 99.37-100.79% and 99.33-100.53%, respectively

**Fig. 2:**
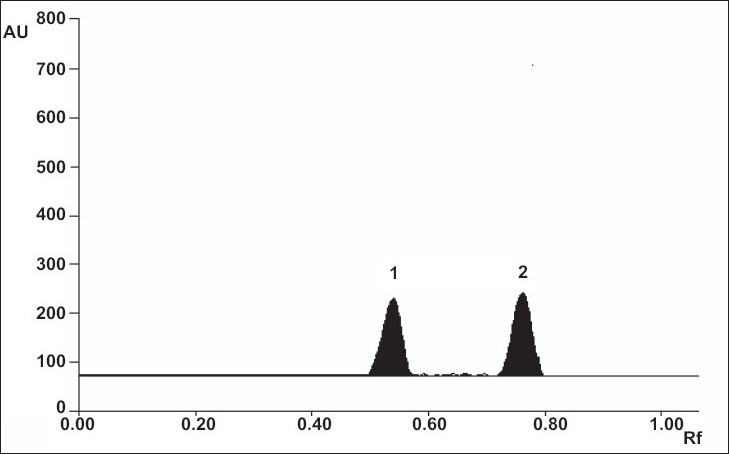
A typical chromatogram of quinapril hydrochloride and hydrochlorothiazide. A typical chromatogram showing quinapril hydrochloride at peak 1, R_f_- 0.51 and hydrochlorothiazide at peak 2, R_f_-0.76.

The proposed HPTLC methods provide simple, accurate and reproducible quantitative analysis for simultaneous determination of quinapril hydrochloride and hydrochlorothiazide in tablets. The reported HPLC method^6^ employed two mobile phases over a reversed phase column coupled with pre-column-solid-phase extraction, to separate the drug from its metabolite, indicating high cost required for such analysis. The linearity and accuracy of the method^6^ are not conforming to the requirements of ICH guidelines.

Moreover, the method reported in present article can be employed for separation of quinapril hydrochloride from hydrochlorithiazide; the drug available in several combination-formulations with quinapril.
